# Innovative Applications of Hydrogels in Contemporary Medicine

**DOI:** 10.3390/gels11100798

**Published:** 2025-10-03

**Authors:** Maciej Rybicki, Karolina Czajkowska, Agata Grochowska, Bartłomiej Białas, Michał Dziatosz, Igor Karolczak, Julia Kot, Radosław Aleksander Wach, Karol Kamil Kłosiński

**Affiliations:** 1 Students’ Scientific Association at the Department of Biomedicine and Experimental Surgery, Faculty of Medicine, Medical University of Łódź, Narutowicza 60, 90-136 Łódź, Poland; maciej.rybicki@student.umed.lodz.pl (M.R.); agata.grochowska@student.umed.lodz.pl (A.G.); bartlomiej.bialas@student.umed.lodz.pl (B.B.); michal.dziatosz@student.umed.lodz.pl (M.D.); igor.karolczak@student.umed.lodz.pl (I.K.); julia.kot@student.umed.lodz.pl (J.K.); 2 Department of Biomedicine and Experimental Surgery, Faculty of Medicine, Medical University of Łódź, Narutowicza 60, 90-136 Łódź, Poland; kczajkowska2701@gmail.com; 3 Institute of Applied Radiation Chemistry, Faculty of Chemistry, Lodz University of Technology, Wróblewskiego 15, 93-590 Lodz, Poland; 4 Biomaterials Research Laboratory, Faculty of Medicine, Medical University of Łódź, Narutowicza 60, 90-136 Łódź, Poland

**Keywords:** hydrogels, medical application, contemporary medicine, drug delivery, abdominal surgery, gynecology, cardiology, rheumatology, urology, ophthalmology

## Abstract

Hydrogels are hydrophilic, soft polymer networks with high water content and mechanical properties that are tunable; they are also biocompatible. Therefore, as biomaterials, they are of interest to modern medicine. In this review, the main applications of hydrogels in essential clinical applications are discussed. Chemical, physical, or hybrid crosslinking of either synthetic or natural polymers allow for the precise control of hydrogels’ physicochemical properties and their specific characteristics for certain applications, such as stimuli-responsiveness, drug retention and release, and biodegradability. Hydrogels are employed in gynecology to regenerate the endometrium, treat infections, and prevent pregnancy. They show promise in cardiology in myocardial infarction therapy through injectable scaffolds, patches in the heart, and medication delivery. In rheumatoid arthritis, hydrogels act as drug delivery systems, lubricants, scaffolds, and immunomodulators, ensuring effective local treatment. They are being developed, among other applications, as antimicrobial coatings for stents and radiotherapy barriers for urology. Ophthalmology benefits from the use of hydrogels in contact lenses, corneal bandages, and vitreous implants. They are used as materials for chemoembolization, tumor models, and drug delivery devices in cancer therapy, with wafers of Gliadel presently used in clinics. Applications in abdominal surgery include hydrogel-coated meshes for hernia repair or Janus-type hydrogels to prevent adhesions and aid tissue repair. Results from clinical and preclinical studies illustrate hydrogels’ diversity, though problems remain with mechanical stability, long-term safety, and mass production. Hydrogels are, in general, next-generation biomaterials for regenerative medicine, individualized treatment, and new treatment protocols.

## 1. Introduction

Modern science has turned to materials with unique physicochemical and biological properties, with the purpose of finding applications in various domains of life and in fulfilling specific needs of industry. Biomaterials, i.e., materials that are intended to be applied in medicine, pharmacy, and related fields, are particularly distinguished because of their exceptional biological properties and interactions with cells and various tissues, which is a critical issue. Load-bearing applications require stiff and durable biomaterials, whereas highly elastic or soft biomaterials are likely to be applied in soft tissue solutions or in tissue engineering. Besides others, one of the requirements that may be advantageous for some tissue engineering applications is the ability to absorb water; therefore, hydrogels (HGs) constitute a noteworthy direction in biomaterials science and an important part of biomedical engineering industry.

Van Bemmelen was the first to use the term “hydrogels” in 1896; however, it referred to the colloidal copper oxide absorbing and retaining ‘a large amount of water’ [1]. The current definition refers to water absorption yet strictly distinguishes the absorbing material. In this view, hydrogel is a hydrophilic polymer network efficiently absorbing and retaining water or physiological fluid, changing its dimensions but still maintaining its structural integrity [2].

The first commercialized use of HGs is considered to be Ivalon^®^ in 1949. It was a sponge-like implant made of foamed polyvinyl alcohol (PVA). Formaldehyde (CH_2_O) was used as the crosslinking agent. Interestingly, it could be freely modeled to achieve the desired shape, making it easy to produce personalized scaffolds with adequate mechanical properties [3]. In addition, various modifications of this HG were developed in the 1950s. One of the most innovative appears to be the addition of heparin, which inhibited thrombocytes adsorption and aggregation, hence prolonging blood coagulation time. Such a property allowed for the use of Ivalon^®^ devices in direct contact with blood, e.g., arteriovenous valves, as demonstrated for dogs, or membranes in artificial kidneys [3]. Another revolutionary example of the commercialized medical application of HGs, is that based on poly(hydroxyethyl methacrylate) (pHEMA) used in contact lenses and orbital implants, as described by Wichterle and Lim in Nature in 1960 [4]. As far back as 1971, Bausch & Lomb’s Soflens product received FDA approval as the world’s first soft HG corrective lenses available in trade [5]. Since then, HGs have been extensively researched for use in agriculture, medicine, pharmaceuticals, and various industries, particularly in the food industry.

It is worth emphasizing that the characteristics of these materials can be easily controlled in the design and production phases, allowing for the creation of ‘tailor-made’ HGs, precisely suited to a specific application. All the changes in the composition, chemistry, and molecular weight of used polymer(s) lead to specific characteristics of the produced hydrogels, of which the common features are porosity, flexibility, and predetermined water content [6]. The aforementioned ability to absorb water is referred to as hydrogel swelling or water uptake. The phenomenon of water absorption proceeds in three stages. Initially, water attaches to hydrophilic groups with the participation of hydrogen bonds (stage 1), then the formed complexes repel each other with hydrophobic groups (stage 2). Free spaces are then formed, which are immediately filled by water unbound to the structure (free water) (stage 3) [7]. Interestingly, HGs can even absorb several hundred times more water than their own weight [8]. The presence of multiple polar groups in the BM structure is seen as the important contributing factor to this property.

Biocompatibility, low immunogenicity, ability to retain and release drugs or other bioactive substances, and programmable biodegradability (if necessary) are the essential properties of hydrogels, predestining them for use in various branches of medicine. Some similarity of HGs to the extracellular matrix (ECM) in mammalian tissues is especially noteworthy. Nevertheless, in contrast to the ECM, conventional hydrogels have limited mechanical resistance and are more susceptible to deformation, particularly under pressure of other tissues, and may be degraded by enzymes present in the body environment [8,9]. To circumvent this disadvantage (for some applications) it is necessary to achieve efficient crosslinking of the constituting polymers.

The categorization of HGs is complex and multifaceted. There are several criteria for the classification of these materials, of which the most common are the origin of the polymer, the structure of the polymer network, the mode of crosslinking, the area of application, and the ability to absorb water (A Comprehensive Review of Hydrogel Classification, Fabrication, and Utility, Amreen Mansuri, Dishant Gupta, Rajat Pawar, Sampat Singh Tanwar, 2025). The ability of hydrogels to swell is in strict correlation with the degree of crosslinking or crosslinking density—the more dense the polymer network, the lower the swelling ratio [10]. These are essentially two mechanisms of crosslinking, physical and chemical, but other means of providing inter-polymer linkages are also possible, such as biochemical (enzymatic) and hybrid methods [11,12,13,14,15]. Chemical crosslinking can be accomplished by the utilization of crosslinking compounds or crosslinkers, which are typically multifunctional monomers, and the reactions are conducted with initiators and/or catalysts, typically at an elevated temperature. As the classical methods are limited because of the presence of toxic compounds, alternative methods involve the irradiation of polymers (or even monomers) in solution, with the ionizing radiation of gamma rays or electron beam, and in some cases, UV light (photoinitiators are required). The advantage of using radiation is the possibility of performing synthesis and sterilization simultaneously, in one technological step [16,17,18]. HGs obtained by chemical methods are stable due to covalent bonds making up the network. On the other hand, physical crosslinking is due to ionic attraction, hydrogen bonds, hydrophobic interactions, or other weak forces. The resulting hydrogels may turn back to sol upon a change in environmental factors, such as pH, temperature, or others [9,19].

The aim of this work is to analyze the development of innovative hydrogels for medical applications, to demonstrate their current use and foreseen perspectives in modern medicine, i.e., in therapeutics, diagnostics, and tissue regeneration, with a special focus on the exceptional properties of these biomaterials. Hydrogels for wound treatment were intentionally omitted, since they had been covered by other reviews. The paper further aims to evaluate the potential of hydrogels as next-generation materials for regenerative and personalized medicine, with an emphasis on their expanding role in contemporary therapeutic strategies and their promise in driving the advancement of future treatment approaches. In the view of the rapid advancements in biomaterial technologies and the as yet incompletely elucidated potential of hydrogels, further comprehensive research is needed. Therefore, this review is intended to highlight the significance of this topic and to stimulate continued scientific inquiry and research activities. Applications of hydrogels in several principal medical areas are presented as follows: gynecology, cardiology, rheumatology, urology, ophthalmology, and surgery.

## 2. Material and Methods

This review was designed as a narrative review of the latest and most innovative applications of hydrogels in selected branches of medicine: gynecology, urology, ophthalmology, oncology, cardiology, abdominal surgery (gastrointestinal tract), and bone and joint diseases (including rheumatology). The purpose was to identify novel directions for the use of hydrogels, especially those that have only recently been introduced into clinical practice or remain at the experimental stage.

### 2.1. Sources of Literature

The literature search was conducted in the following databases: PubMed, Scopus, Web of Science, Elsevier, and Google Scholar. Gray literature, conference reports, and patents were not included. Only peer-reviewed articles with full text and abstracts available in English were included in the analysis.

### 2.2. Search Strategy

The basic keywords were variations in the terms hydrogel(s) and biomaterial(s), combined with terms specific to individual specialties. Queries were constructed using the logical operators AND and OR. Examples of keyword sets included:

Gynecology: “Hydrogel(s)” AND “Gynecology”; “Hydrogel(s)” AND “Uterus”; “Hy-drogel(s)” AND “Ovarian cancer”; “Hydrogel(s)” AND “Cervix”; “Hydrogel(s)” AND “En-dometriosis”; “Hydrogel(s)” AND “Contraception”; “Hydrogel(s)” AND “Vaginal infec-tions”; “Hydrogel(s)” AND “Sexually transmitted infections”; “Hydrogel(s)” AND “Pelvic inflammatory disease”; “Hydrogel(s)” AND “Vaginal drug delivery”.

Urology: “Hydrogel(s)” AND “Urology”; “Hydrogel(s)” AND “Bladder”; “Hydrogel(s)” AND “Urinary tract cancers”; “Hydrogel(s)” AND “Kidney”; “Hydrogel(s)” AND “Prostate”.

Oncology: “Hydrogel(s)” AND “Oncology”; “Hydrogel(s)” AND “Tumor therapy”; “Hydrogel(s)” AND “Chemotherapy delivery”; “Hydrogel(s)” AND “Immunotherapy”; “Hydrogel(s)” AND “Radiotherapy”.

Cardiology: “Myocardial infarction” AND “Hydrogel(s)”; “Cardiomyocytes” AND “Hydrogel(s)”; “Myocardial infarction” AND “Cardiac Patch”; “Cardiac regeneration” AND “Hydrogel(s)”.

Abdominal surgery/gastrointestinal: “Abdominal surgery” AND "Hydrogel(s)”; “General Surgery” AND "Hydrogel(s)”; “Hernia Surgery” AND "Hydrogel(s)”; “Intestinal adhesions” AND “Hydrogel(s)”; “Abdominal trauma” AND “Hydrogel(s)”.

Bone and joint diseases/Rheumatology: “Rheumatoid arthritis” AND “Hydrogel(s)”; “Rheumatoid arthritis” AND “Hydrogel(s)” AND “Drug medium”; “Rheumatoid arthritis” AND “Injectable hydrogel(s)”; “Rheumatoid arthritis” AND “Oral hydrogel(s)”; “Rheumatoid arthritis” AND “Hydrogel(s)” AND “Tissue scaffolds”; “Rheumatoid arthritis” AND “Hydrogel(s)” AND “Immunomodulator”; “Rheumatoid arthritis” AND “Hydrogel(s)” AND “Lubricant(s)”.

Ophthalmology: “Hydrogel(s)” AND “Ophthalmology”; “Hydrogel(s)” AND “Cornea”; “Hydrogel(s)” AND “Retinal disease”; “Hydrogel(s)” AND “Drug delivery eye”; “Hydrogel(s)” AND “Ocular implants”.

The queries were tailored to the specific characteristics of each database, but their overall structure remained consistent. The search was not limited to titles and abstracts alone—full texts were also included in order to increase the chances of identifying valuable publications.

### 2.3. Inclusion and Exclusion Criteria

All types of peer-reviewed studies (original papers, reviews, in vitro studies, in vivo studies, and clinical trials) were included in the analysis, provided that they were available in English and had the full text available. However, the following articles were excluded:

- Those published in a language other than English;
- Those without an abstract or full text;
- Editorial comments, letters to the editor, or introductory notes.

In accordance with the objective of the study, priority was given to innovative applications of hydrogels, which is why greater emphasis was placed on research published in recent years (the exact time frame varied depending on the field discussed).

### 2.4. Selection Process

The selection of articles was carried out by at least two independent reviewers. First, the titles and abstracts were analyzed, followed by the full texts. In the event of discrepancies, the decision was made through discussion, and if this did not resolve the issue, the decision was made by a third reviewer.

### 2.5. Data Synthesis

The articles included were reviewed in a descriptive manner. No formal meta-analysis or statistical synthesis of data was performed, as the primary aim of the study was to present and discuss new directions for the application of hydrogels in various fields of medicine.

## 3. Properties and Applications of Hydrogels in Medicine

### 3.1. Hydrogels in Gynecology

Hydrogels are used in gynecology for the production of contraceptive implants, wound dressings, and drug delivery systems, as well as in experimental treatments for cervical insufficiency. The increasing number of new articles discussing the applications of these materials is recorded; however, most of the applications do not consider hydrogels as the first choice for treatment.

Injuries of the endometrium—the inner lining of the uterus—can have significant clinical implications, particularly in the context of fertility and reproductive health. Such injuries may result from surgical procedures (e.g., curettage, hysteroscopy), infections, chronic inflammation, or mechanical trauma. One of the most serious complications is Asherman’s syndrome, characterized by intrauterine adhesions that can lead to infertility, menstrual irregularities, or recurrent pregnancy loss. Proper regeneration of the endometrium is essential for embryo implantation and the maintenance of pregnancy, so any damage to this tissue requires careful diagnosis and appropriate treatment. One of the methods to treat the problem is using hydrogels scaffolds. The main objectives of applying hydrogel in endometrial therapy are to prevent the formation of adhesions, repair damaged endometrial tissue, and promote its regeneration. Key factors considered when selecting suitable hydrogel matrices include their biocompatibility, biodegradability, mechanical properties, lack of immunogenicity, and ability to support endometrial healing and restore reproductive function, as well as their capacity for controlled drug release. Two key features of hydrogel systems are their low immunogenic potential and biomimetic adequacy with the tissue to be healed. Minimal immune response allows both the hydrogel and incorporated therapeutic agents to remain in the body without being targeted as foreign substances, thereby enabling them to effectively reach and act on the site of injury. Therefore, hydrogels are used for endometrial regeneration because they also offer a highly supportive environment that replicates natural tissue, simultaneously delivering therapeutic agents [20,21].

Miconazole is one of the best examples in gynecology of switching a drug formulation from a 2% cream or vaginal suppositories to a hydrogel. It is recognized that irregular or insufficient application of creams has often led to treatment failure and recurrence of the infection. Due to its advantageous structure and strong adhesive properties, the hydrogel—forming polymers such as Carbomers or Hydroxyethylcellulose—provide therapeutic concentrations of a medicine incorporated in its structure. Therefore, it is better suited for application on the skin because it provides prolonged release of the active substance due to inherent ability to retain water and adhere to the skin surface—these also enhances drug penetration. In addition, hydrogels offer a cooling and soothing effect reducing irritation and, moreover, enhance and help maintain skin hydration, thus the use of hydrogels improves patients’ comfort. Antibacterial hydrogel-forming polymers such as Poloxamers or Carbomers are also useful in the treatment of bacterial infections, but with the usage of antibiotics [22].

Hyaluronic acid (HA) may be used in the form of a hydrogel. If it is properly crosslinked, it offers durability and prolonged absorption. HA hydrogels are very often applied in the treatment of atrophic vaginitis [23]. Bacterial vaginosis, with a prevalence ranging from 10% to 40% among women aged 14–49 years, represents a major gynecological concern due to its high recurrence rate and potential reproductive health implications. The use of chlorhexidine-based vaginal hydrogel has been proposed as a therapeutic approach, providing both antimicrobial activity and restoration of vaginal microbial balance, thereby addressing key aspects of disease management [24]. As demonstrated by the examples, hydrogels are effective carriers for various substances that are advantageous for treatment of vaginal disorders.

A recent development can be credited to researchers of ETH Zurich and Empa, who have prevented endometriosis with a hydrogel implant. The proposed hydrogel system is composed of two acrylamide-based polymers crosslinked with either the photolabile molecule poly(ethylene glycol) diacrylate or the disulfide crosslinker N,N’-bis(acryloyl)cystamine; therefore, it can be degraded on a clinically relevant time scale by near-visible UV light or any disulfide-reducing agents, such as the biocompatible glutathione (1). The hydrogel material allows the device to be placed anywhere in the fallopian tube, as it is initially small and expands by absorbing water, thereby isolating the two ends of the tube. It provides a very soft and durable structure which ensures effective sealing. The hydrogel can be easily removed, which offers an option for women who do not want to use hormonal contraception or cannot use it due to medical contradictions. There has not yet been an evaluation of hydrogel implant stability over a long time period once placed in the fallopian tubes, especially during intense physical activity; therefore, further studies are necessary [25].

#### Intravaginal Treatment of Fungal and Bacterial Infections

**Figure 1:**
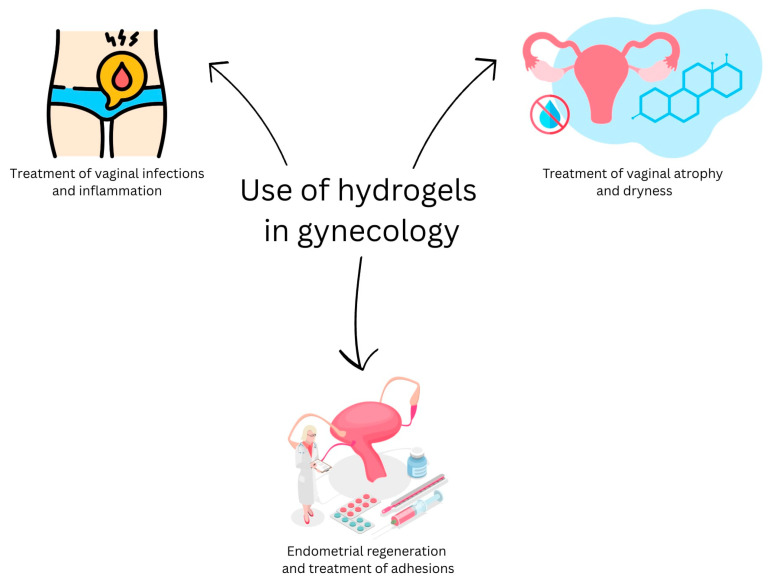
Hydrogel use in gynecology.

Recent trends clearly demonstrate a scientific interest in the application of hydrogels in gynecology, as evidenced by the increasing number of related publications. This surge in research highlights the promising potential of hydrogel-based systems in addressing various gynecological conditions, particularly in areas such as endometrial regeneration, adhesion prevention, targeted drug delivery, and fertility preservation. Many studies have shown that hydrogels not only offer biocompatibility and controlled release properties but also support tissue repair and immune modulation. Given their versatility and ability to be tailored for specific therapeutic needs, hydrogels are regarded as a future cornerstone in gynecological regenerative medicine and personalized therapy. The Figure 1 shows selected applications of hydrogels in gynecology.

### 3.2. Hydrogels in Cardiology

Myocardial infarction causes significant damage to both the structure and function of the heart. Even though the human body possesses the ability to generate cardiomyocytes, they are insufficient for repair [26]. Ischemic heart disease (IHD) has been the most significant cause of health loss worldwide for the last few decades. There were ca. 7.3 million cases of acute myocardial infarctions (MIs) in 2015 [27]. Therefore, the life-saving strategies and further treatment for IHD are continuously under development, some involving hydrogels.

**Figure 2:**
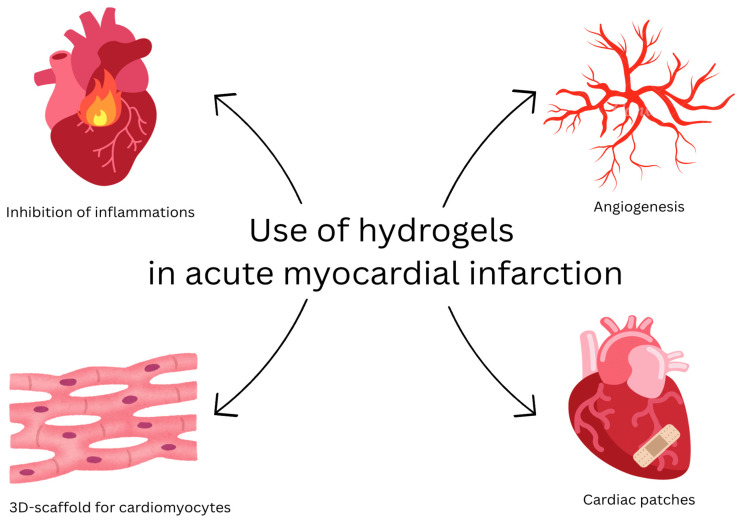
Hydrogel use in acute myocardial infarction (created in Canva).

The first report on hydrogel use in myocardial infarction treatment comes from 1995. However, it was focused on hydrogel-coated balloons and delivery of other active agents via catheters [28,29]. Some of the first uses of hydrogel materials that acted directly in the cardiac muscle were described in 1998 and 2001. The first study examined the use of heparin-alginate devices containing basic fibroblast growth factor (bFGF) and their influence on the collateral development of cardiac muscle [30]. The second study evaluated whether hydrogel microspheres impregnated with basic fibroblast growth factor could promote collateral development post-MI. In this study, the material was administered subepicardially [31]. The aim of hydrogel-based treatment of MI is to reduce myocardial injury by inhibiting inflammatory response [32], promote angiogenesis [33], or apply a tissue engineering approach to create a three-dimensional scaffold for cardiomyocytes’ seeding and proliferation in vitro, to be subsequently implanted into myocardium [34]. An alternative approach is the implantation of cardiac patches, which are hydrogel structures placed onto the surface of the heart. Cardiac patches are well integrated with the organ, which allows not only for protection but also adequate conduction [35]. Said patches should possess mechanical properties suitable for structural support of the myocardium and should allow for cardiomyocyte alignment similar to that of functional cardiac tissue. Their electrical conductivity helps prevent reperfusion arrhythmias [36]. The Figure 2 shows selected applications of hydrogels in cardiology.

Currently, the treatment of MIs focuses on reperfusion and pharmacological therapy. The latter concentrates on the treatment of heart failure, lowering LDL level, antithrombotic regimens, and anti-inflammatory therapy. The regenerative approach to MI treatment remains in the development stage, although it has not yet reached large-scale clinical trials [37]. The regenerative aspect focuses, for example, on miRNA, cell cycle regulators, and the employment of stem cells [38]. Nevertheless, these treatment options do not consider the use of hydrogel materials as a primary vector for the delivery of active agents [39,40,41].

There are various materials being assessed for functionality: the potential negative or positive outcomes of their use. Examples below illustrate the fulfillment of MI treatment objectives. A hydrogel was developed from the decellularized ventricular ECM, which was tested on rats. The ECM was processed into an injectable liquid that self-assembled upon injection. MI was induced and the hydrogel was injected into the myocardium 2 weeks later. The results showed an increase in cardiomyocytes, although they did not present a statistically significant heart performance increase. The material mimicked the native cardiac extracellular environment [42]. Gel@MSN/miR-21-5p, which was an injectable hydrogel material carrying microRNA-21-5p, was produced by mixing the MSN/miR-21-5p solution with an aqueous solution of α-CD (cyclodextrin) and aldehyde-capped polyethylene glycol. The study showed that the pro-angiogenic and anti-inflammatory effect of the method reduced MI size in a porcine model. The material’s degradation in vitro was tested. In phosphate-buffered saline (pH of 6.8) it lost 93% of its initial mass. In vivo measurements using a fluorescence signal revealed signal decay to 16% of its initial value on day 14. However, the material allowed for preservation of the ventricular wall thickness in the infarcted area [43]. A melanin nanoparticles/Alginate hydrogel was proven to reduce oxidative damage to cardiomyocytes and induce macrophage polarization to the M2 type, which supports myocardial regeneration. This material was proven to be able to provide the appropriate mechanical support needed for cardiac repair. The surface of the material was rough, which could promote the adhesion of cardiomyocytes to the hydrogel at the infarction site, enhancing the repair effect. In vivo degradation was tested and resulted in ca. 20% hydrogel weight loss after 14 days of observation. The test was performed on a rat model and showed statistically significant results [44]. An injectable mechanical–electrical coupling hydrogel patch was created that inhibited dilation of the MI-affected area, assisted cardiac function, and improved electrical conduction and synchronization. The hydrogel consisted of gelatin, PVA (polyvinyl alcohol), and b-PANi (bentonite-polyaniline) [45]. Nano-films were formed by depositing PLL (poly L-lysine)-coated graphene oxide sheets onto cell layers. Cardiomyocytes inside the construct showed a high level of proliferation and cell viability. The tissue showed spontaneous beating and actuation under a low-level external electric field [46]. Other materials include a chitosan–glutathione-based injectable hydrogel that suppressed oxidative stress damage in cardiomyocytes [47] and a tunable self-healing ionic hydrogel (POG1) which, similarly to the melanin nanoparticles/Alginate hydrogel, possessed mechanical properties suitable for use in the MI environment. It showed promising cardiomyocyte orientation results as a cardiac patch [48].

Possible methods of cardiac hydrogel implantation include injection into the myocardium [49] and cardiac patches implanted onto the epicardium that provide mechanical support and release bioactive agents using microneedles. However, this method can be invasive, as it may require a thoracostomy to be performed [50]. Materials can be designed in such a way that an intrapericardial injection is possible—hydrogel has been injected into the pericardial cavity, with a minimally invasive procedure. It formed a cardiac patch directly on site [51]. In some cases, an injection may not be needed. Paintable hydrogels—soft, flexible, and sticky paste-like hydrogels— are viscous enough to attach themselves to the heart surface, forming a patch-like structure in situ [52].

From these examples, an outline of hydrogel materials used can be derived. Materials can be both of natural origin (decellularized ventricular ECM) and synthetic (POG1). Some possess the ability to sustain a prolonged release of the active agent (Gel@MSN/miR-21-5p). Some are nano-films that form larger structures, whereas others only contain nanoparticles. If designed for injection, their structure should allow passage through the needle without destruction by the shearing forces or forming the gel in situ. The hydrogel material is biocompatible and stable enough to withstand the constant mechanical strain of cardiac work. The application of ionic conductive hydrogels allows for the MI-affected area to become a conductive pathway for electrical signals. These materials can be used to suppress oxidative stress and modify inflammatory response locally rather than through systemic action, as in the classical methods of MI treatment. In addition, hydrogels can alter their characteristics based on environmental conditions.

Regarding hydrogel-based drug delivery systems for myocardial infarction, there are various obstacles that should be overcome. One of them is the continuous movement of the heart. The substance can be washed away via venous drainage or squeezed out of the myocardium [53,54]. It is worth noting that MI is a continuous process, and the timing of injection is likely to have a significant influence on therapeutic success [55]. There are three main phases of AMI: the inflammatory phase, during which cardiomyocyte death occurs; the proliferative phase, which is marked by the resolution of inflammation and myofibroblast proliferation; and the maturation phase, during which the ECM of scar tissue becomes crosslinked and myofibroblasts deactivate. Each of these phases has a certain time span [56]. It is thought that elevated activity of collagenase and gelatinase during the inflammatory phase may result in the deterioration of collagen-based hydrogel materials [57]. A study performed by Blackburn et al. showed that injecting collagen matrix into a mouse heart at 3 h post MI has significantly prevented ventricular remodeling in comparison to 2 weeks and 3 weeks post MI [58]. Injection of hydrogel into the myocardium naturally comes with a dilemma of choosing the proper material volume. A study performed on swine showed that 2 and 4 mL injections into the MI site have a more positive effect on LV remodeling than 1 mL [59]. Another study proved that injection volume did have a significant effect on the mechanical resistance of myocardium. However, it also showed that precise injection depth is not necessary [60]. As proven by Dileep D et al., the mammalian heart cardiomyocytes show longitudinal orientation with more complex orientation at the apex [61]. It has already been proven that three-dimensional engineering of cardiac tissue is possible. However, it was looser than its natural equivalent [62]. A “Frame Hydrogel” methodology has been invented to generate cardiac tissue with highly mature functional properties. It possesses diverse 3D geometry and structural and functional anisotropy. Said tissues can mature quickly without the need for complex stimulation [63]. When it comes to injectable hydrogels, three ways of delivery can be outlined. The material can be delivered intracoronary, epicardially, or transendocardially. It has to gel quickly at the site of MI, but not prematurely, to avoid catheter blocking. It should be biodegradable, and it has to mechanically support the ventricular wall [64].

As hydrogels can be designed in such a way that they change their properties when stimulated, they seem to be advantageous for MI treatment. As proven by the following studies, several stimuli, both natural and artificial, can be used to influence hydrogel-based implants. It is important to add that natural ECM does undergo dynamic changes in response to various stimuli, and therefore so should hydrogel-based materials. For example, their stiffness may be modulated by light, pH, or temperature [65]. As the infarct microenvironment is acidic, the pH-responsive hydrogels deposited into the myocardium can deliver bioactive agents locally. In the study by Cheng N et al., the hydrogel response to weakly acidic pH allowed for the controlled release of metformin and exosomes that improved cardiac repair. Their material was developed by crosslinking oxidized hyaluronic acid and carbohydrazide-modified collagen. It demonstrated self-healing properties and elasticity similar to the myocardium [66]. Temperature-responsive hydrogels loaded with a desired substance allow for its controlled release [67]. The material ability to change phase from liquid to gel at a certain temperature may allow for better injectability. An example of this property is a hybrid hydrogel scaffold of aminated guaran and lipid extracellular matrix [68]. Hydrogels sensitive to reactive oxygen species (ROS) can be designed to release bioactive agents if stimulated by the presence of ROS. A hydrogel created by UV light-induced polymerization of monomers synthesized from ethylene glycol sulfur acrylate is an accurate depiction of ROS reactivity. ROS influenced the drug (5-fluorouracil) release from the hydrogel and, interestingly, induced material swelling [69]. As demonstrated by Zhi Zheng et al. in a rat MI model, a ROS-responsive hydrogel released liposomes loaded with ROS scavengers and thus improved cardiomyocyte function [70]. Ultrasound-activated hydrogels delivered into the tissue gelled on site, thus overcoming the barrier of tissue penetration. A study by Zhao proved that a fibrin hydrogel network can be formed by the ultrasound-triggered release of thrombin from liposomes. In addition to that, said materials may stimulate micro-vascularization [71].

To deliberate on the clinical applications of hydrogels in MI, one needs to know how research has progressed. The vast majority of research involves animals. The reports prove that hydrogel-based treatment, combined with other therapeutic options, does effectively restore cardiac function and preserve its morphology [51,72]. The studies that have involved the use of hydrogel-based materials in MI patients remain a small fraction of all research in this subject. The clinical trials sought involve an insignificant number of patients; however, they indicate that hydrogel MI treatment is possible, and hydrogels are well tolerated and do have a positive effect on cardiac function [49,73]. Drawing a direct comparison between classical and hydrogel-based intervention in humans poses a challenge, as those two approaches have different indications and outcomes.

To summarize, there are numerous ways to approach hydrogel-based MI treatment. Animal and preliminary human tests have proven that hydrogels are a feasible option that could be used to improve existing treatment plans, thus increasing the effectiveness of the intervention. There are still obstacles to be tackled, such as collagenase activity or timing of intervention. An important issue is that of on-site gelation, which allows for easy injection, simultaneously rendering the material mechanically resistant on site. In 2024, 71 research papers were published (on Pubmed) regarding hydrogel use in MI. They focused on inhibiting inflammatory response, angiogenesis [49], the inhibition of oxidative stress [74] and exosome use [75]. One of the most prominent aspects of research is the development of self-healing hydrogels that can repair themselves when damaged. This allows for a more native environment in comparison to non-healable hydrogels [76].

The use of hydrogels in the treatment of MI is an ever-expanding field of research. One can only speculate what the future will bring, both to scientists and to patients. This part of the review clearly depicts the numerous possible implementations of hydrogel-based materials in the treatment of myocardial infarction. Moreover, it shows that they might be the future of cardiology and regenerative medicine altogether.

### 3.3. Hydrogels in Bone and Joint Diseases

#### 3.3.1. Rheumatoid Arthritis

Rheumatoid arthritis (RA) is currently the most frequently diagnosed inflammatory arthritis. It causes permanent disability and shortens life expectancy by 6–7 years, alongside causing reduced quality of life [77,78]. It can be defined as a chronic, autoimmune, inflammatory joint disease resulting in both articular as well as extra-articular complications affecting the cardiovascular, pulmonary, digestive, and nervous systems. RA typically presents as bilateral joint stiffness, swelling, and pain, as a consequence of synovitis. Due to its progressive character and periodical exacerbations, cartilage and bone degenerate over time, causing irreversible deformities [79,80].

Although the etiology remains unknown, a fundamental role seems to be played by defective citrullination, leading to the production of anti-citrullinated antibodies (ACPAs) [81]. The ACPAs can activate macrophages and osteoclasts, promoting bone loss alongside inflammation. Furthermore, some human leukocyte antigen (HLA) variations are responsible for environmental interaction between smoking and higher risk of the disease, establishing an additional vulnerable population [79].

Existing forms of modern RA treatment include physical therapy, drug management, and in some instances surgical procedures. The main pharmaceutics used are non-steroidal anti-inflammatory drugs (NSAIDs), steroids, conventional and biologic disease-modifying, antirheumatic drugs (DMARDs) such as methotrexate, leflunomide, sulfasalazine, and chloroquine, and in severe cases, biological agents slowing RA evolution and inducing remission [82]. This approach focuses on reducing inflammation and provides remission or relatively low disease activity, simultaneously frequently causing major adverse effects as a result of pharmacotherapy [83]. Consequently, studies concerning the improvement of RA management have been carried out. Among them, the potential use of hydrogels proved to be promising.

#### 3.3.2. Hydrogels in Rheumatoid Arthritis

**Figure 3:**
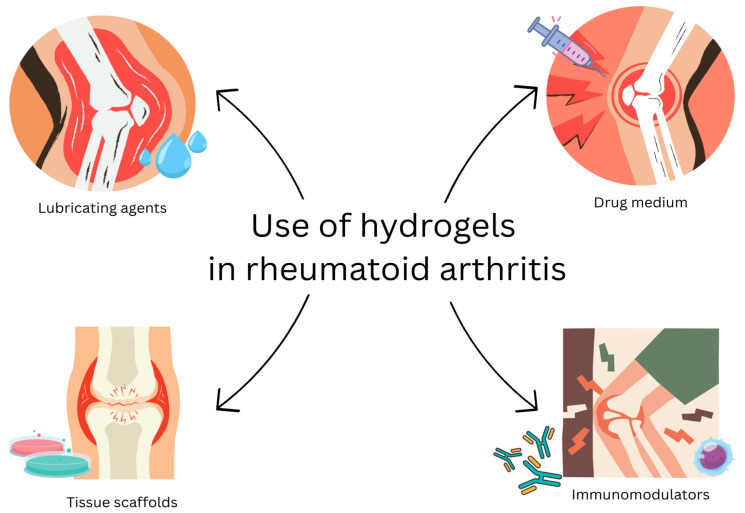
Hydrogels in RA (created in Canva).

Since 1991, 157 articles have been published on PubMed concerning the use of hydrogels in RA, about 45 of them in 2024 alone. Currently, there are four main ways in which the potential use of hydrogels could be beneficial in the treatment of RA, such as drug medium, tissue scaffolds, lubricating agents, and immunomodulators [83]. Among them, using hydrogels as drug mediums has been reported the most. The Figure 3 shows selected applications of hydrogels in bone and joint diseases.

Certain characteristics of hydrogels to be applied in RA treatment are especially desired, namely, biocompatibility, safety, and possession of suitable mechanical and rheological properties enabling durability in articulation. The implementation of foreign materials into the human body involves the issue of biocompatibility. Subsequently, the composition of hydrogels is adjustable to be tolerable in the organism and have properties satisfying the anticipated application, making it possible to imitate living tissues [84]. Materials used for manufacturing hydrogels for RA therapy include natural polymers such as hyaluronic acid, proteins, chitosan, and alginate, and synthetic ones, mainly PVA and PEG. All of these materials are biocompatible and some of them possess antimicrobial properties, decreasing the risk of infection and immune rejection while increasing the time during which the hydrogel can remain inside the tissues [83]. Since the treatment of rheumatoid arthritis is a long-term process, all the properties of hydrogel extending its ability to remain in the human body are highly crucial.

Due to hydrogels’ mechanical, rheological, and fatigue properties, they can be injected into the articulation, posing as synovial fluid, tissue scaffolds, immunomodulators, and drug delivery medium, decreasing the risks of systemic treatment necessity. As mentioned before, hydrogels comprising polymers in three-dimensional structures are perfect matrices or carriers for the encapsulation of drugs and secondary substances [83,85].

Alongside the issue of biocompatibility, the injection of gel may also pose technical difficulties. Consequently, thermo-sensitive hydrogels that are soluble at room temperatures and solidify at body temperature [85] facilitates the injection and does not tend to migrate to adjacent tissues, further localizing the treatment. Another approach has been made using photopolymerization, manufacturing hydrogels that are initiated in lower light intensities through free radical polymerization [86].

#### 3.3.3. Hydrogels as Drug Medium

The main advantage of injecting pharmaceuticals by means of hydrogels into the synovial cavity is omitting the first-pass effect, which would be present in oral drug application, as well as easily reaching proper therapeutic concentration. Furthermore, due to specific application along with controlled drug release [83], the risk of main adverse effects of pharmacotherapy is decreased. Pharmaceuticals that can be injected via hydrogel medium include NSAIDs, DMARDs, steroids, and TNFα inhibitors.

A study on rats showed that injecting methotrexate by means of self-forming hydrogels into the articular cavity leads to the local release of anti-inflammatory H_2_S gas while reducing NO production, aiding in healing inflammatory bone erosion. Moreover, due to the component of hyaluronic acid, which is an integral part of cartilage, additional lubrication was provided by the hydrogel, alleviating the symptoms and reducing the number of injections needed [87]. This study highlights how crucial it is for the management of chronic diseases to be less time consuming and less unfavorable to the quality of life. Additionally, one property of HA, namely, the ability to form lubricating complexes with phospholipids, developed cartilage cushioning, reducing articular abrasion. This property inspired another study on mice where a nanomedicine hydrogel based on HA was created and used to deliver celastrol into the joint. It resulted in bone regeneration and a decrease in inflammation by means of crosstalk of fibroblasts and macrophages [88].

Another in vivo and in vitro study examined mediating drugs through double layered, hydrogel transdermal patches made with hyaluronic acid, dexamethasone, and naringin, an antimicrobial agent. Said patches were able to release 98% of the contained dexamethasone during the course of 10 days. As reported, levels of inflammatory cytokines (IL-6, TNF-α) were reduced, alleviating the RA symptoms and preserving joint structure [89]. However, some pharmaceuticals comprise larger molecules, restricting their skin absorption, favoring injectable hydrogels but limiting their use as a transdermal medium. Still, extending the period between the next applications to 10 days remains a remarkable feature, and if applied in future, it would have a positive outcome on the quality of life of patients.

There were also reported preclinical trials focusing on the encapsulation of oral medicine hydrogel. Not every oral drug requires such countermeasures, but therapeutic antibodies, due to their vulnerability to acidic pH and stomach enzymes, do. As of today, biotherapeutics are mainly administered through subcutaneous, intravenous, or intramuscular injections. Thus, manufacturing a more accessible smaller-dose, oral route may be beneficial. This particular study examined pH-responsive microparticle hydrogels as a transmucosal delivery system, minimizing degradation while preserving the bioactivity of biotherapeutics. The hydrogel was designed in such a way to release antibodies in neutral pH, simulating a small intestine environment [90], further highlighting the role of customization. Further developments concerning the use of antibodies via hydrogel medium in RA are anticipated.

#### 3.3.4. Hydrogel Lubricating Properties

As synovitis is the main component of RA, protecting and regenerating the synovium plays a vital role during therapy. The synovium lubricates joint cartilage, maintains non-adherent tissue surface allowing for movement, and controls balanced levels of synovial fluids. In early stages of RA, the synovium thickens and ratio between different chondrocytes building the synovium changes, disrupting the microarchitecture alongside joint function [91]. However, as the disease progresses, inflammation destroys the synovium and the volume of synovial fluid decreases, causing cartilage and bone destruction [83].

Hydrogels made with enriched hyaluronic acid were designed to lubricate and to regenerate cartilage in RA. According to the report, dopamine-, SO_3_^2−^- and kartogenin-enriched injectable hydrogel proved to have bioadhesive and lubricating properties, not only preventing cartilage degradation through an anti-inflammatory and anti-oxidative environment, but also repairing the damage caused by late-stage RA both in vitro and in rat models [92]. This may prove beneficial in aiding treatment even in severe cases of RA, highlighting again that hydrogels have potential in this field.

#### 3.3.5. Tissue Scaffolds

Hydrogels can mimic cartilage properties, protecting affected joints [83]. This feature may alleviate pain, reducing the role of standard pain pharmacotherapy with its adverse effects, and delay the negative impact of disease on quality of life. Because cartilage has a limited ability to regenerate, an increasing number of studies are exploring cartilage tissue engineering using hydrogels [83]. The use of synthetic polymers, such as PEG and PVA, may ensure high mechanical properties needed to withstand the forces present in joints. However, typically, hydrogel scaffolds based on densely crosslinked synthetic polymer have a compression modulus below 1000 kPa, which is lower than that of natural cartilage, leaving room for improvement. Nevertheless, hydrogels can be manufactured to create a suitable environment for chondrocytes to develop and to produce the extracellular matrix in order to strengthen the hydrogel, aiding in withstanding compressive forces [93].

#### 3.3.6. Immunomodulators

Immune response through ACPAs and rheumatoid factor (RF) may be present long before clinical symptoms of RA set in. Both are linked to severe course of the disease and joint destruction. Thus, exploring hydrogels’ possibility of immunomodulating has been investigated. This characteristic is achieved by the addition of mesenchymal stem cells (MSCs) to the injectable hydrogel.

Immunomodulatory hydrogels have been reported to influence the local joint microenvironment by neutralizing pro-inflammatory cytokines, thus modulating the immune response [83]. A study from Haotian Bai et al. questioned whether a bone marrow mesenchymal stem cell (BMSC)-loaded 3D injectable hydrogel could boost the performance of osteoblasts, enhancing bone regeneration. A 3D inorganic–organic supramolecular bioactive interface was created by combining stiff, porous metal scaffold, injectable polysaccharide supramolecular hydrogel, and immunomodulating agents such as BMSCs and bone morphogenetic protein 2 (BMP-2). The study demonstrated that BMP-2 could maintain its metabolic function as well as a sustainable release from hydrogel, inducing osteogenesis and decreasing osteoporotic microenvironment in vivo and in vitro [94].

Numerous studies involving the use of hydrogels in rheumatoid arthritis emerge each year, further indicating the possible future treatments using hydrogels and their properties. Certain difficulties, such as high manufacturing costs and the structure and size of certain pharmaceuticals, need to be considered and overcome. Furthermore, hydrogels are easier to manufacture in smaller quantities, as it is feasible to maintain precise composition, restricting their wider use. Even though the materials used are biocompatible, the issue of long-term internal stability has not been tested clinically [83].

#### 3.3.7. Summary

To conclude, hydrogels may be a revolutionary way of treating chronic arthritis including RA, covering most of the pathogenesis aspects. They can be tailored to possess certain mechanical and rheological properties, enabling durability in articulation. For more sophisticated requirements, they can be manufactured for a more personalized treatment, covering individual patient needs. So far, the described properties and the results of hydrogels testing have proven them to be promising materials for rheumatoid arthritis.

### 3.4. Hydrogels in Urology

The application of hydrogels in urology is a relatively new innovation in biomedicine. The first report on modern hydrogel materials used to construct ureteral stents was released in 1988 [95]. Just a year later, it was shown that coating catheters with hydrogel exhibiting antimicrobial effects could prevent infections [95]. More than 30 years later, urinary tract infections remain a significant clinical challenge, and, to date, no material has been developed to fully prevent infections in catheterized patients.

In 2024, according to the PubMed database, as many as 111 scientific articles on the use of hydrogels in urology have been published, more than double the number published in 2020. This dynamism demonstrates the growing interest of the academic community and the considerable potential that HGs show in the treatment of urological conditions.

**Figure 4:**
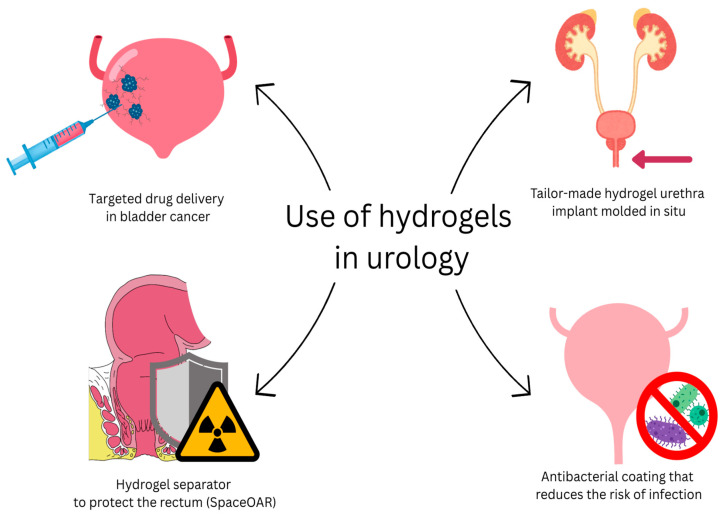
Summary of the use of hydrogels in urology (created in Canva).

**Figure 5:**
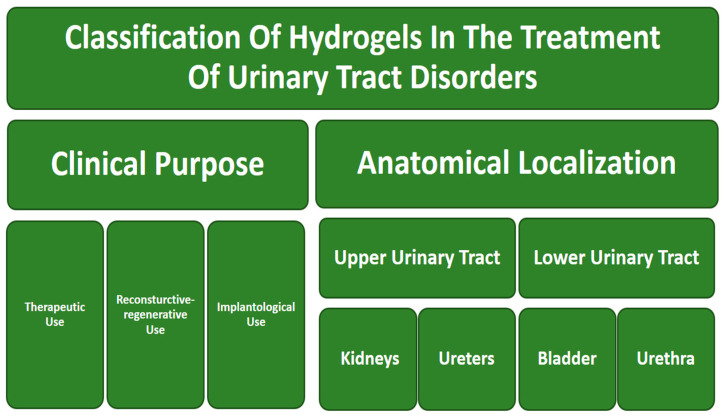
Classification of hydrogels in the treatment of unitary tract disorders.

Due to unique physicochemical properties, hydrogels have increasing application potential in the treatment of urinary tract disorders. There has been noticeable growth in the scientific community’s interest in this matter in recent years. However, it should be emphasized that this area is still considered relatively new, and the complete potential of hydrogel-based materials in urology requires further in-depth exploration. This chapter presents an arbitrary classification of hydrogel applications in urology, according to clinical purpose and anatomical localization. The most straightforward and intuitive way to classify hydrogel applications in the treatment of urinary tract disorders is for clinical purposes. Three major sub-categories can be distinguished here, i.e., therapeutic use, reconstructive–regenerative use, and implantological use (Figure 5). An alternative, no less valued approach is to classify by the anatomical location in which the HG material is used. In this view, there is a general division into the upper and lower urinary tracts, or a more detailed one distinguishing kidneys, ureters, bladder, and urethra (Figure 5) [96]. Such an overview simplifies the analysis of the available solutions and directions of biomaterials development in urology. The Figure 4 shows selected applications of hydrogels in urology.

Urinary tract cancers are among the most common malignant tumors in men in Poland. They affect approximately one in three men diagnosed with cancer. In 2020, more than 37,000 new cases of these cancers were registered. The leading cancers are prostate cancer (20%), bladder cancer (6.7%), and kidney cancer (3.7%). Moreover, as many as 20% of cancer-related deaths in males are due to urinary tract cancers [97]. The treatment techniques for these neoplasms, in view of current medical achievements, are apparently inadequate, so more sophisticated therapeutic methods are being applied, e.g., intravesical drug delivery systems. It is possible to inhibit tumor growth by directly depositing a hyaluronic acid hydrogel matrix with iron oxide nanoparticles onto the surface of the malignant tissue. This provides for long-term and sustained dosing of the drug, which increases the uptake of IONPs within the carcinoma cells by up to 50 times while maintaining in-depth penetration into the tissue. It should be emphasized that this method omits crucial organs, that is, it employs targeted therapy in the case of chemo-resistant bladder cancer [98].

Another promising implementation of HGs in prostate cancer radiotherapy is a hydrogel separator (SpaceOAR Hydrogel, Figure 5). While conventional treatment methods such as radiotherapy are often effective, they unfortunately may lead to complications resulting from irradiation of the perineal area, especially the rectum. Polyethylene glycol-based HG (PEG) can function as a temporary spatial barrier between the prostate and rectum [99]. This solution significantly reduces the radiation dose reaching the rectal tissue walls, reducing the risk of inflammation, hemorrhage, diarrhea, or chronic pain. Furthermore, it also allows for the application of higher therapeutic doses while maintaining low toxicity, improving the safety and efficacy of the treatment [100].

Properly crosslinked hydrogels can mimic the mechanical properties of urethral cells. An example of such a material is alginate hydrogel, crosslinked with a mixture of calcium chloride and bromine chloride. Trials were carried out in a solution mimicking artificial urine, in order to reflect the target environmental conditions in which the material would function [101]. The results demonstrated that the hydrogel maintained its mechanical and structural properties for up to 12 weeks. Due to its biodegradability, it was subsequently degraded in the simulated environment. Such mechanical and degradable characteristics make the alginate hydrogel a highly prospective material for use as a biodegradable ureteral stent [101].

Alginate hydrogel with poly(2 hydroxyethyl methacrylate) (p-HEMA), crosslinked by visible light, demonstrates potential for use in the recovery of urethral lacerations. Significantly, this material can be molded using a portable 3D printing pen, allowing for the precise design of any shape according to the individual needs of the patient. Such a solution becomes particularly important when there is considerable anatomical variation in the urethra, which is challenging and induces additional risk when using conventional reconstruction methods [102]. Furthermore, 3D printing pen technology is applicable in replication of irregular and complex lesions, thus creating a tailor-made three-dimensional reconstructive structure that matches both anatomically and functionally with damaged or substituting tissue [102].

Moreover, p-HEMA hydrogel with the addition of selected fatty acids, in particular myristic acid and decanoic acid, demonstrated adhesion-reducing properties for bacteria, including the most common urological pathogens such as Escherichia coli, Proteus mirabilis, and Staphylococcus epidermidis [103]. The ability to reduce microbial adherence represents significant potential for further research into the use of this material in the prevention of urinary tract infections. In particular, its future use as an antimicrobial coating for the surface coating of urinary catheters seems promising, which may help to bring down the risk of infections associated with their application [103].

**Figure 6:**
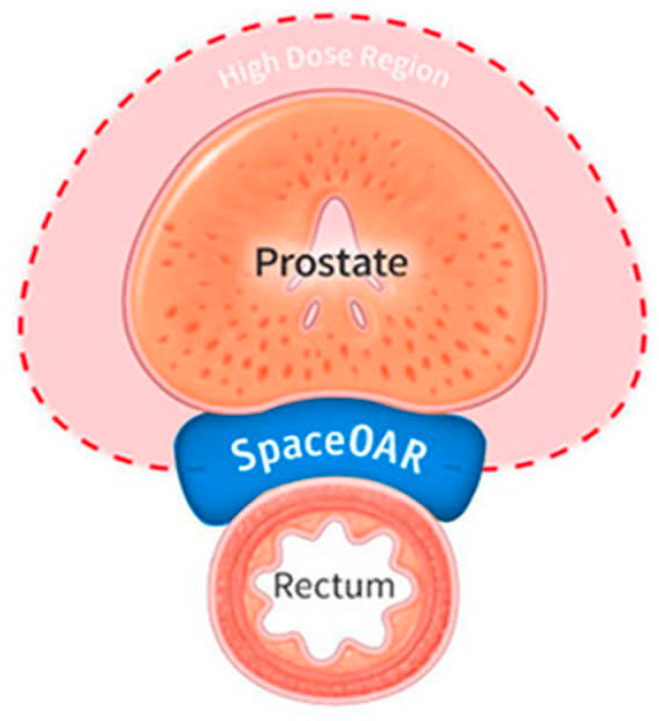
Location of the SpaceOAR hydrogel [99]. “Image provided courtesy of Boston Scientific. © 2025 Boston Scientific Corporation or its affiliates. All rights reserved.”

A patch made from polyvinyl alcohol (PVA) has proven potential for use in procedures targeted at increasing penile size. In studies carried out on an animal model (rabbits), high efficiency was noted, with no evidence of material degradation nor fibrosis of the surrounding tissue. Advantageously, the material retained appropriate mechanical durability and in vivo stability during long-term follow-up [104]. This example demonstrated that not only can life-threatening diseases or injuries in the urological field be remediated with hydrogels, but also some dysfunctionalities may be treated as well. The results suggest that PVA may find expanding applications in tissue engineering, specifically in the post-surgical reconstruction of tissue and organs after the surgical removal of tumors. Due to its flexibility, biocompatibility, and ability to be specifically designed, the material also shows potential for use in the beauty and esthetics industry [104]. Figure 6 shows the exact location of the SpaceOAR hydrogel.

#### Summary

New hydrogel applications in urology, such as cancer treatment, tissue regeneration, and placement as implants, are under development. Besides facilitating therapeutic targeted drug delivery to the bladder, HGs serve as protective barriers in prostate radiation therapy and promote the healing and reconstruction of the urethra. The biomedical urology offered by modern technologies such as 3D printing, as well as the biodegradability and antimicrobial effects of hydrogels, make them highly interesting materials for further development.

### 3.5. Hydrogels in Cancer Therapy/Oncology

**Figure 7:**
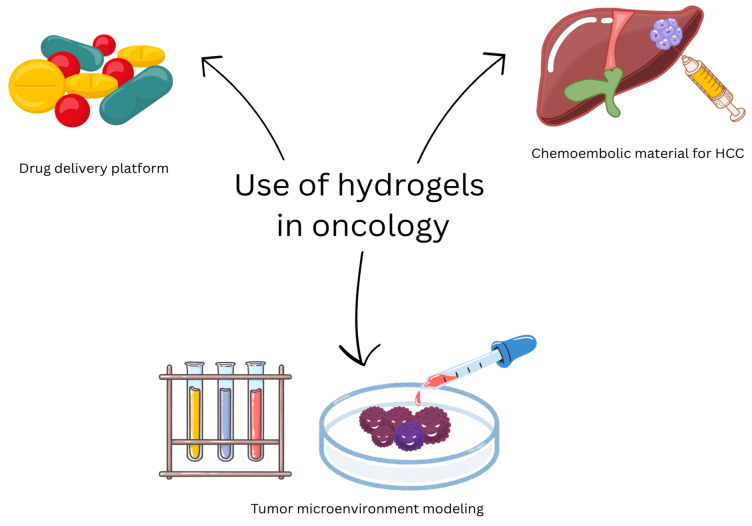
Summary of the use of hydrogels in oncology (created in Canva).

According to GLOBOCAN 2020, cancer ranks as the second leading cause of death worldwide—in 2020, there were approximately 19 million new cases and 10 million deaths from cancer [105]. Despite technological advances, classic treatment methods often have serious side effects and limited effectiveness due to complex interactions in cancer tissue that are still not fully understood. For this reason, there is a constant need to develop new solutions that will facilitate cancer therapy through a better understanding of in vivo conditions and intercellular interactions within the tumor. One of the directions is polymer hydrogel materials, which are finding increasingly wider application in oncology. This section discusses the current and potential applications of hydrogels in the context of cancer therapy. The Figure 7 shows selected applications of hydrogels in oncology.

#### 3.5.1. Hydrogels as Drug Delivery Systems and Modifiers of Drug Release

Cancer treatment is frequently associated with numerous side effects resulting from the low selectivity of conventional cytotoxic drugs and the inability to control the site and rate of their release. Combined therapies are particularly problematic, as they are associated with an even higher risk of toxicity [106]. In response to these challenges, efforts are being made to develop modern drug delivery systems, among which hydrogels stand out as carriers enabling controlled and localized delivery of anticancer drugs [107]. Research is progressing on the use of hydrogels in the delivery of classic cytostatic drugs like doxorubicin and platinum-based drugs, e.g., cisplatin and carboplatin. There are two main classes of these materials: synthesized, ready-to-use hydrogels that must be surgically implanted, and hydrogels that form in situ, which can be injected with minimal invasiveness in liquid form and then gelate under the modulation of endogenous stimuli such as temperature, pH, or ion concentration. The drug incorporated in the hydrogel matrix is released by diffusion-controlled kinetics or triggered by specific environmental conditions [108]. These systems allow for the controlled, prolonged release of drugs at the tumor site, which ensures a high local concentration and therefore reduces systemic toxicity. Studies have shown that hydrogels (including those based on alginate, gelatin, chitosan, amphiphilic triblock hydrogel PLGA-PEG-PLGA, and copolymer mPEG-b-PELG) with doxorubicin are more effective against cancer than traditional forms of delivery (mainly systemic), while reducing side effects [109]. In addition, hydrogels can effectively deliver and release hydrophobic drugs, improving their solubility and stability, which distinguishes them favorably from conventional administration [110]. Hydrogels, e.g., those based on chitosan, alginate, or polyacrylic acid, sensitive to a stimulus, such as pH changes or the presence of enzymes characteristic of the tumor microenvironment, are suitable for the targeted release of platinum-based drugs, further increasing the precision of therapy and reducing undesirable effects [111].

In addition, an advanced hydrogel prepared from CS-dextran phosphate carbamate enables the simultaneous delivery of doxorubicin (DOX) and indomethacin (IND). IND, used as an analgesic and anti-inflammatory drug, has a synergistic anticancer effect of reducing the expression of the MRP1 transporter, which increases the concentration of DOX in cancer cells and enhances its anticancer activity. Hydrogels based on dextran phosphate carbamate allow for the prolonged release of DOX and the delayed release of indomethacin, which enhances the therapeutic effect in in vitro cancer models [112].

One of the best-documented examples of the clinical use of a hydrogel as a local delivery system for anticancer drugs is Gliadel plate. This biodegradable therapeutic system consists of polifeprospan 20 copolymer, a hydrogel carrier, impregnated with carmustine (1,3-bis(2-chloroethyl)-1-nitrosurea (BCNU))—an alkylating cytotoxic drug. Several small implants are placed in the cavity left after the removal of the glioblastoma, where they gradually release the drug over several weeks. The BCNU acts locally within the tumor remnants. This form of local treatment avoids the blood–brain barrier permeability factor, which limits the effectiveness of systemic chemotherapy in the brain. Phase III clinical trials have shown that the use of Gliadel implants significantly prolongs median overall survival (OS) in patients with either recurrent or newly diagnosed glioblastoma multiforme (GBM). Gliadel implants have been approved by the FDA for use since 1997 [113].

There are high expectations regarding the use of hydrogels in nanomedicine, particularly nanotheranostics. Intensive research is being conducted on the role of poly(acrylic acid)-based nanogel as a delivery system for radioisotopes. This nanogel is engineered with Lys1Lys3-bombesin(1–14) modified with the chelator 1,4,7, 10-tetraazacyclododecane-1,4,7,10-tetraacetate (DOTA), enabling the precise transport of radionuclides such as Y-90 and Lu-117, which are applied in prostate cancer treatment [114]. Bombesin has a role as a ligand for the gastrin-releasing peptide receptor (GRPR), a cell surface receptor that is overexpressed on prostate cancer cells [115]. DOTA stabilizes the radioisotope and prevents its release in tissues without GRPR expression. In in vitro tests, the nanoparticles showed high precision in targeting tumor cells, which significantly increases the effectiveness of cancer therapy. However, in vivo studies in rats showed that distribution in tumor cells was below expectations, because the radioisotope accumulated to a large extent in the liver, a common issue observed with many nanoparticle-based carriers. Thus, the use of these nanogels requires further research to improve biodistribution and reduce side effects [114]. Hydrogels as anticancer drug delivery systems are currently at various stages of development, ranging from preclinical research to clinical application. Stimuli-responsive hydrogels such as alginate, PLGA-PEG-PLGA, hyaluronic acid, or PCLA-PEG-PCLA with PAMAM dendrimers acting as drug carriers, that respond to environmental stimuli, e.g., ionic interactions, temperature, and pH, are currently in the experimental phase, where their efficacy, biocompatibility, and drug release profiles are being evaluated. At the same time, there are examples of hydrogels already implemented in medical practice, such as Gliadel plates used in the treatment of gliomas, which confirms their practical use as effective and safe drug carriers in cancer therapy [107,108].

#### 3.5.2. Hydrogels as Platforms for Modeling Tumors and Their Microenvironment

One of the key challenges in developing new therapeutic strategies in oncology is the need for reliable assessment of the anticancer activity and toxicity of new chemical compounds or drugs in the preclinical stages. Two-dimensional (2D) cell culture models are most often used for this purpose, as these models are widely available and standardized. However, they have significant limitations: they do not reflect the complex tumor microenvironment present in vivo, including interactions between tumor cells and stromal cells, blood vessels, and the extracellular matrix [116,117]. For this reason, 2D models often yield unrealistic predictions of tumor response to treatment. It is estimated that only about 4% of compounds demonstrating anticancer activity in vitro successfully complete four phases of clinical trials [118]. This discrepancy between preclinical results and clinical efficacy highlights the necessity to develop more representative in vitro models that better mimic the structure and function of tumors in vivo.

Polymer hydrogels, owing to their physicochemical and biological properties, such as their ability to create three-dimensional networks, their high water content, and the possibility of their precise modification, represent an interesting platform for remodeling the complex tumor microenvironment. They can be engineered with regard to elasticity, crosslinking density, bioactivity, and degradability, enabling the creation of in vitro environments that support tumor cell growth, migration, and interactions with the extracellular matrix [119]. Moreover, the ability to precisely control the mechanical properties of hydrogels, especially stiffness, is crucial for replicating tumor microenvironment conditions. Hydrogels’ stiffness as a scaffold affects the functioning of cancer cells, their invasiveness, and their response to treatment. By adjusting this property, different stages of tumor progression can be simulated, even recreating the microenvironment characteristic of malignant tumors, rich in ECM [120]. An example of utilizing such hydrogel properties is the application of photopolymerizable poly(ethylene glycol) diacrylate (PEGDA) with the addition of methacrylated gelatin (methagel) to create self-folding, curved microstructures that mimic the architecture of mammary gland ducts. Differences in the swelling behavior of two PEGDA layers allow for controlled curvature formation, while methagel as a component of the outer layer improves tumor cell adhesion to the resulting structure. They can be employed for long-term culture of cancer cells, including breast cancer models, serving as platforms for imaging, drug testing, and studying tumor growth dynamics in in vitro conditions [121].

In recent years, hydrogels have often been used to research crucial biological processes for cancer, such as invasion, metastasis, angiogenesis, and the impact of matrix stiffness and hypoxia on tumor cell behavior. They are used in research on brain tumors [122], to better understand the tumor microenvironment, as well as prostate and breast cancer, where the molecular mechanisms responsible for tumor growth and metastasis are evaluated [117,123]. These models are valuable tools in screening tests for the effectiveness of anticancer drugs, allowing for a better understanding of the mechanisms of disease progression and supporting the development of personalized therapies [121].

#### 3.5.3. Chemoembolic Materials in the Treatment of Hepatocellular Carcinoma

One of the methods used to treat hepatocellular carcinoma (HCC) is transcatheter arterial chemoembolization (TACE), which aims to occlude the hepatic artery and cut off the blood supply to the tumor, delivering chemotherapy locally. The embolization materials used so far (e.g., Lipiodol, Gelfoam) have significant limitations, such as short duration of action (<3 days), heterogeneous properties, and the risk of systemic toxicity [124]. In response to these challenges, efforts are being made to develop new and safer embolization materials, including polymer hydrogels. Of particular interest are, again, hydrogels with the ability to change phase under the influence of environmental stimuli (e.g., pH), allowing the material to be administered as a liquid and converted to a solid at the site of the pathological lesion [125].

One example is amphiphilic anionic PCLA-PUSSM copolymer made of poly(ethylene glycol) (PEG), poly(ε-caprolactone-co-lactide) (PCLA), and poly(urethane sulfide sulfamethazine) (PUSSM), which was used in studies on VX2 rabbit hepatocellular carcinoma models. They demonstrated the ability to rapidly change phase from liquid to solid at low pH, leading to the effective embolization of tumor blood vessels and enabling the controlled release of doxorubicin (DOX). The use of these hydrogels also allowed for maintaining high embolization efficacy and sustaining therapeutic drug concentrations in the tumor for several weeks, while reducing the volume of injected material [124].

Additionally, hydrogels can be enriched with contrast agents, e.g., PCL-PEG-based material enriched with Lipiodol, a X-ray long-lasting contrast agent, to facilitate their localization and monitoring during the procedure [125].

Another example is IF@Gel hydrogel made of Poloxamer-407 hydrogel, idarubicin hydrochloride, and Fe_3_O_4_ nanoparticles, which was examined in rat models. It demonstrated effective inhibition of tumor growth, induced strong necrosis and apoptosis of cancer cells, and enabled long-term drug release at the tumor site compared to traditionally used chemoembolization materials. Moreover, the Fe_3_O_4_ nanoparticles contained in the hydrogel allowed for the precise monitoring of the therapy using magnetic resonance imaging. Moreover, the local presence of the hydrogel induced no detectable systemic toxicity [126]. Hydrogels used in TACE are still in the preclinical research stage, where experiments were conducted mainly on animal models, such as rats and rabbits, with orthotopic liver tumors. However, the results obtained so far indicate their significant therapeutic potential. The properties presented constitute a promising area for the development of modern therapeutic materials in the treatment of HCC, but their introduction into clinical practice still requires many stages of scientific research [124,126].

#### 3.5.4. Summary

The use of hydrogels in cancer therapy is an interesting and promising area of research aimed at improving the effectiveness of cancer therapy and reducing its side effects and toxicity. A search on PubMed for the terms “hydrogel” and “oncology” yields 275 results from the last 5 years. Most applications of hydrogels in oncology are still in the preclinical stage, e.g., platforms for precise drug delivery stimulated by environmental stimuli, nanogels for the delivery of radioisotopes, or hydrogels acting as embolization agents, and require further research to assess their safety, biodistribution, and drug release control. In clinical practice, however, Gliadel plates are used to deliver carmustine in the treatment of gliomas. Hydrogels are also used in research on the tumor microenvironment, where they act as a scaffold replacing the ECM for cancer cells. The results of this research are closer to reality than studies on 2D cell cultures and offer promising prospects for further developments in oncology.

### 3.6. Hydrogels in Ophthalmology

**Figure 8:**
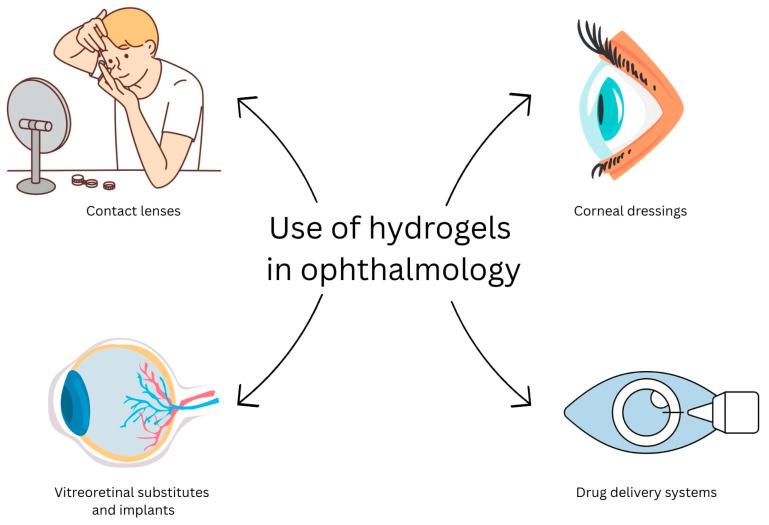
Hydrogels for ophthalmology classification [128].

The World Health Organization (WHO) estimates that eye diseases affect nearly 260 million people worldwide. Among them, as many as 86% are patients suffering from visual impairment, whereas the remaining 14% are blind. The most common vision disorders include glaucoma, cataracts, and age-related macular degeneration (AMD). It is worth noting that, according to the Central Statistical Office, every second adult in Poland has a sight impairment [127]. The first documented use of a hydrogel in ophthalmology was in contact lenses in 1960—it was made of HEMA [4]. Since then, the number of possible applications of this material has grown steadily. A search in PubMed for the terms “hydrogel” and “ophthalmology” yields 809 papers in the last 5 years alone. In 2025, there were already 139 results in the period up to July. Hydrogels intended to be used in ophthalmology can be most simply classified based on their anatomical application (Figure 8):

· Intended for anterior segment of the eye:
  ○ Contact lenses;
  ○ Corneal dressings;
  ○ Drug delivery systems for the eye surface;
  ○ Adhesive hydrogels for wound sealing;
  ○ Intraocular implants (IOLs).
· Intended for posterior segment of the eye:
  ○ Vitreous substitutes;
  ○ Drug delivery systems for the vitreous.

Contact lenses are now widely available and extremely popular among doctors and patients. There are two main types of lenses, i.e., hydrogel-type based on polymers, such as p-HEMA, PVP, and poly(methyl methacrylate), and hydrogel-silicone type, utilizing those hydrophilic polymers and polydimethylsiloxane or other silicone polymers. The former provides better hydration, constantly moisturizing the cornea; they are very thin and flexible. However, over time, water evaporates, resulting in poorer oxygen permeability to the cornea [129]. Conversely, lenses with the addition of silicone improve oxygen transport to the cornea due to their enhanced porosity. This structure, however, has a notable downside. It promotes the accumulation of contaminants and protein deposits. Therefore, even greater attention must be paid to hygiene when wearing them, which can be problematic for everyday use [130]. The widespread use of lenses with hydrogel additives has established their primary role in vision correction and, besides hydrogel dressings for burn wounds, is unprecedented in the use of hydrogels for medical purposes in general.

Corneal bandages are used after cataract or glaucoma surgery, as well as in cases of corneal epithelial defects or after corneal transplantation. Their purpose is to provide protection against external factors, ensure an optimal environment for healing wounds, and relieve pain after surgery. With the appropriate additive, they can also promote the regeneration and repair of the corneal epithelium [128,129]. Clinical trials are currently underway on hyaluronic acid and gelatin-based hydrogel bandages Kuragel, which are expected to revolutionize corneal transplants. Such transplants are extremely rare at present due to significant differences between donors. Corneal transplants are threatened by infections, lack of cohesion, and the need for surgical stitching of the resulting wounds. Kuragel is a replacement solution capable of promoting scar-free regeneration of the epithelium and stroma [131]. Studies have shown that the hydrogel stimulates the growth of epithelial cells in the upper layer and stromal cells in the inner mass. These results were confirmed in experiments conducted on rabbits. Densitometry and pachymetry measurements show restoration of corneal transparency, optical density, and thickness, comparable to those observed in a healthy cornea, within two months after application of the hydrogel [131].

A common, but very dangerous condition faced by patients is retinal detachment that leads to irreversible blindness. Current best practice is to remove the vitreous body and replace it with another material [132,133]. Nevertheless, no material currently available clinically can be used as a long-term substitute for the vitreous body. Sulphur hexafluoride, perfluorocarbon gases, or silicon-based oils are used temporarily [133]. Hydrogels are an interesting alternative to the materials used so far, especially poli(vinyl alcohol) (PVA) and polyethylene glycol (PEG). The injection of a hydrogel solution significantly reduces the invasiveness of the procedure compared to the classical method. The refractive index, viscosity, stability, optical transparency, and mechanical properties of the hydrogel biomaterial are similar to those of the natural vitreous body [134].

Stimuli-responsive hydrogels also find their use in ophthalmology, particularly in the treatment of dry eye disease (DED). DED leads to neurosensory disorders, as well as eye surface or lacrimal gland inflammation. The basic method of DED treatment is the use of artificial tears or infrared eye masks. However, both of those methods are impractical for everyday use [135,136]. An alternative may be mini eye patches made of photothermal gelatin and gold nanotubes, which are attached to the lacrimal gland. Their task is to convert light radiation into heat energy and thus stimulate the lacrimal gland to produce more tears. Studies have also confirmed the protective properties for the eyes [137].

The given examples demonstrate that applications of hydrogels in ophthalmology have a long history and are well established; however, new solutions involving hydrogels are under investigation, as the soft hydrophilic polymer networks are particularly suited to the wet eye environment.

### 3.7. Hydrogels in Abdominal Surgery

The use of hydrogels in abdominal surgery is relatively new. The first reports were published in 1997. In 1999, the first attempt for surgical application was revealed in a preliminary report on the use of hydrogels in rabbits to prevent intra-abdominal adhesions [138]. Intra-abdominal adhesions are still a challenge for modern surgery. It is still a serious complication, often requiring a second operation. In the past five years, 145 articles on research into hydrogels targeting abdominal surgery were published, according to PubMed, which demonstrates sound interest in this topic.

Hydrogels may potentially have many applications in abdominal surgery. Due to their high structure-related biocompatibility and softness, they are less likely to cause material-mediated inflammation and inevitably promote the healing of internal organ damage post surgery. Considering newer applications of hydrogels in surgery, Janus hydrogel, which has an asymmetric structure expressing opposing properties, can be in the form of patches, where one side is anti-adhesive, whereas the other promotes healing, encouraging tissue integration. Zhang et al. introduced a Janus zwitterionic hydrogel patch based on polysulfobetaine-type zwitterion (SBMA) crosslinked with methacrylated hyaluronate acid (HAMA). This basic hydrogel matrix is non-adhesive and biodegradable. One side of the patch was treated to form an adhesive polymer brush layer of poly(acrylic acid-co-N-hydroxysuccinimide acrylate) to promote one-sided tissue adhesion and integration, subsequently accelerating healing. In rat models, histology examination revealed proper healing, minimal inflammation in tissues, and almost complete prevention of adhesion [139]. The material was proven to be strong, but also flexible for in vivo use, and degraded completely within 28 days.

Mehmood et al. presented Janus patches that include zinc-incorporated mesoporous silica nanogranules (ZnMSGs) reinforced catechol-modified gelatin methacrylate (GC) hydrogel. Such a hydrogel adheres easily to the wounded tissue and can be used in internal wounds without adhesions [140].

Janus hydrogel patches can be successfully used in intestinal injuries and anastomoses. Li et al. described a PVA/GA-PAA hydrogel patch made out of polyacrylic acid (PAA) and gelatin (GA) that can be wet-adhered to the surface of the intestine and polyvinyl alcohol (PVA) that prevents adhesions based on the physical barrier. In the rat model, the PVA/GA-PAA hydrogel promoted healing in intestinal injury with no post-operative tissue adhesion [141]. Still, the clinical feasibility in humans needs to be assessed.

Another Janus hydrogel form is injection. Wu et al. presented an injectable photocurable catechol-grafted hydrogel (HAD) and demonstrated its feasibility for covering gastrointestinal perforations and intra-abdominal adhesions [142].

Janus hydrogels can also be sprayed, making them feasible for gastroscopy and gastrointestinal perforations. Wang et al. presented a sprayable Janus hydrogel obtained through modifications of photocurable hyaluronic acid (HA) hydrogel with dopamine and phenylboronic acid, which enabled the formation of reversible boronate ester bonds. In vivo studies with rat models with gastrointestinal perforation demonstrated the efficacy of such sprayable hydrogel in clinical settings [143].

The use of hydrogels in inguinal hernia surgery is not a procedure of choice. As tension-free techniques with prosthetic meshes exist, hydrogels have rather limited applicability. Hydrogels have several disadvantages that limit their use in abdominal surgery, such as poor mechanical strength and rapid deterioration of key properties. In light of such disadvantages, hydrogels with greater durability and hardness may have applications in hernia surgery. The standard prosthetic mesh for hernia surgery is polypropylene mesh (PPM). This type of mesh is mechanically strong, stable, and durable, and can be easily adjusted. However, it is non-biodegradable and has poor biocompatibility. It can lead to foreign-body inflammatory reactions, and due to inflammation, can lead to complications such as abdominal adhesions [144]. Trials to modify PP mesh to become less irritant and non-adhesive involve coating with a hydrogel layer.

Chitosan-based hydrogel-coated mesh is a concept that was introduced to reduce inflammatory response, prevent adhesions, and improve biocompatibility. Chitosan, a natural polysaccharide composed of continuous glucosamine with some N-acetyl glucosamine monomers, has already been used in many appliances due to antibacterial [145,146], hemostatic [147], nontoxic [148], and anti-adhesive/adhesive properties [149,150,151]. Hydrogels can display both anti-adhesive properties by forming a hydrated barrier that prevents protein and cell attachment, and adhesive properties when functional groups (e.g., amines, catechols, aldehydes) enable bonding with tissues, making them versatile for biomedical applications. However, the main disadvantage is its water insolubility [152]. To overcome this, hydroxypropyl chitosan, a water-soluble derivative of chitosan, has been employed. Hu et al. described a photosensitive hydroxypropyl chitosan azide (AZ-HPCTS) hydrogel that underwent UV grafting or crosslinking to the PP mesh. The hydrogel degraded slowly over 180 days in rats. The adhesion scores were significantly lower in the coated mesh group than in the group with non-coated mesh. Also, in the process of defect repair, mesh with AZ-HPCTS promoted wound healing by inducing the secretion of TGF-β1 in the acute reaction stage [153]. However, the studies on humans are necessary to confirm the properties of AZ-HPCTS.

#### Summary

**Figure 9:**
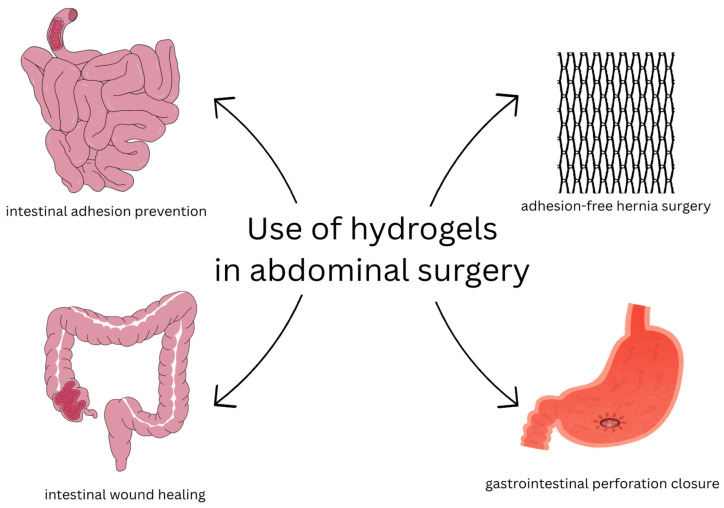
Summary of the use of hydrogels in abdominal surgery (created in Canva).

Hydrogels application in abdominal surgery is still to be developed, but two clinical approaches are under consideration so far. Janus-type hydrogels are a particularly promising form, as they have an asymmetrical structure, with one side preventing postoperative adhesions while the other promotes tissue regeneration. They can be administered in the form of patches, injections, and spray, and have been demonstrated to be effective in treating internal wounds, particularly in gastrointestinal perforation models. The other approach involves inguinal hernia surgery, where the hydrogel is used as coating for regular hernia mesh to reduce inflammatory response and the risk of adhesions. However, further preclinical and clinical studies are needed to assess the effectiveness of these solutions in humans. The Figure 9 shows selected applications of hydrogels in abdominal surgery.

## 4. Conclusions

The representative examples above give an overview of the versatility of hydrogels and their importance for medicine. Generally, polymers forming the network are responsible for mechanical and rheological properties, whereas water provides an appropriate environment of healing and a permeable medium for encompassed therapeutics. Various attributes, such as stimuli-responsiveness or degradability, required for specific applications, are driven by the polymer type. The polymer chemistry determines the properties of the network, and those properties are reflected in adequate applications.

The advantages and the importance of polymeric hydrogels for medical applications are broadly recognized in the literature. The number of scientific publications covering the medical utilization of hydrogels has been increasing and totaled ca. 2200 for the last five years, as presented in Table 1. It should be noted that the hydrogels based on synthetic hydrophilic polymers make up the majority. Among specific fields of medicine, ophthalmology is in particular focus—over 800 papers have been published since 2020. This is mainly due to two inherent features of hydrogels: softness and hydration, which perfectly harmonize with the eye environment. Softness and adherence are critical in the application of hydrogels in either myocardial infarction treatment or urology; both subjects were the topic of 350–400 publications in the five-year period. A pronounced increase has been recorded in the latter case, of which one of the reasons may be the anti-infectious features of hydrogels carrying appropriate active substances. Research on applications of hydrogels in abdominal surgery and rheumatoid arthritis, with less than 150 papers each in the last 5 years, are more common research topics than applications in gynecology. In each of those three areas, softness is again the key property, but adhesion or, conversely, its absence, may be advantageous, depending on the particular function that the hydrogel serves. In RA, injectable gels or in situ forming gels in particular are most used. Research on hydrogels for oncological purposes are steadily growing, and besides direct applications for healing, works on tumor cells containing hydrogel being a model for cancerous tissue are important part of that.

Future hydrogel research will involve the concept of multifunctional and stimuli-responsive hydrogel systems, with the potential to provide personalized, adaptive therapies. New advances in nanotechnology, 3D bioprinting, and bioactive molecule incorporation will further open their potential for regenerative medicine, organ engineering, and precision cancer medicine. The central challenges over the next few years will be to improve long-term stability, provide biocompatibility under mixed physiological conditions, and enable large-scale cost-effective production to hasten clinical translation.

## Figures and Tables

**Table 1:** Number of scientific publications on applications of hydrogels in selected fields of medicine, released in the years 2020–2024, according to the PubMed database. The search keywords were ‘hydrogel’ and the name of the particular field as listed in the table.

	2020	2021	2022	2023	2024	TOTAL
**Gynecology**	8	14	23	16	26	87
**Myocardial infarction**	65	64	74	73	87	363
**Rheumatoid arthritis**	19	22	17	27	45	131
**Urology**	55	60	86	83	111	395
**Oncology**	38	49	51	59	78	275
**Ophthalmology**	134	170	150	148	207	809
**Abdominal surgery**	12	18	28	28	56	142
**TOTAL**	331	397	429	434	610	2202

## Data Availability

No new data were created in this study. Data sharing is not applicable to this manuscript.

## Abbreviations

The following abbreviations are used in this manuscript:

HG	Hydrogel
PVA	Polyvinyl alcohol
pHEMA	Poly(hydroxyethyl methacrylate)
FDA	Food and Drug Administration
ECM	Extracellular matrix
HA	Hyaluronic acid
IHD	Ischemic heart disease
MI	Myocardial infarctions
bFGF	Basic fibroblast growth factor
LDL	Low-density lipoprotein
α-CD	α-Cyclodextrin
PLL	Poly L-lysine
bPANi	Bentonite-polyaniline
ROS	Reactive oxygen species
RA	Rheumatoid arthritis
ACPAs	Anti-citrullinated antibodies
HLA	Human leukocyte antigen
NSAIDs	Non-steroidal anti-inflammatory drugs
DMARDs	Disease-modifying, antirheumatic drugs
RF	Rheumatoid factor
BMSCs	Bone marrow mesenchymal stem cells
BMP-2	Bone morphogenetic protein 2
IONPs	Iron oxide nanoparticles
PEG	Poly(ethylene glycol)
p-HEMA	Poly(2 hydroxyethyl methacrylate)
DOX	Doxorubicin
IND	Indomethacin
BCNU	Carmustine
OS	Overall survival
GBM	Glioblastoma multiforme
GRPR	Gastrin-releasing peptide receptor
PEGDA	Poly(ethylene glycol) diacrylate
HCC	Hepatocellular carcinoma
TACE	Transcatheter arterial chemoembolization
PCLA	Poly(ε-caprolactone-co-lactide)
WHO	World Health Organization
DED	Dry eye disease
SBMA	Zwitterionic sulfobetaine methacrylate
HAMA	Hyaluronic acid
ZnMSG	Zinc-incorporated mesoporous silica nanogranules
GC	Gelatin methacrylate
PAA	Polyacrylic acid
PPM	Polypropylene mesh
BM	Biomaterial
